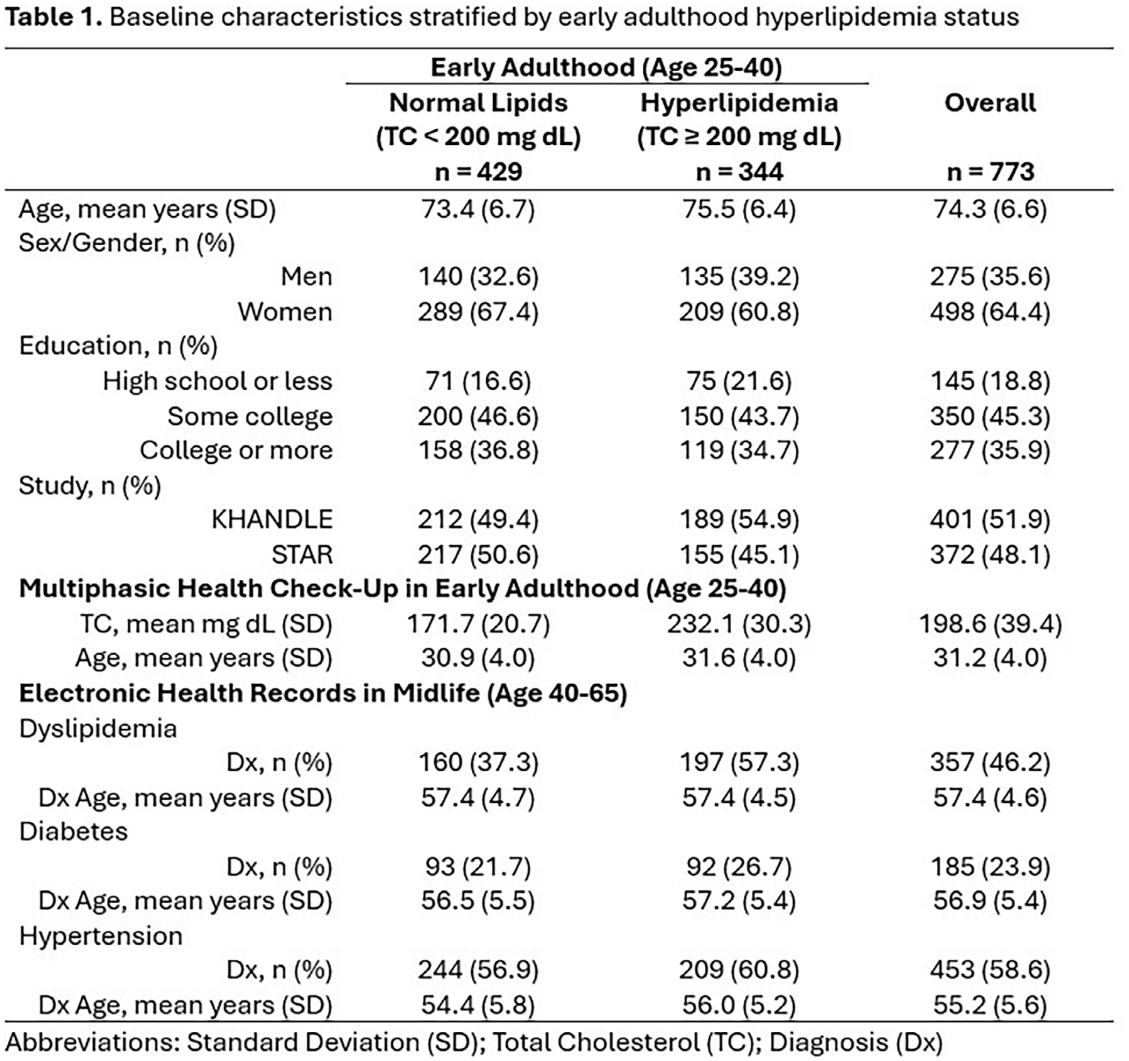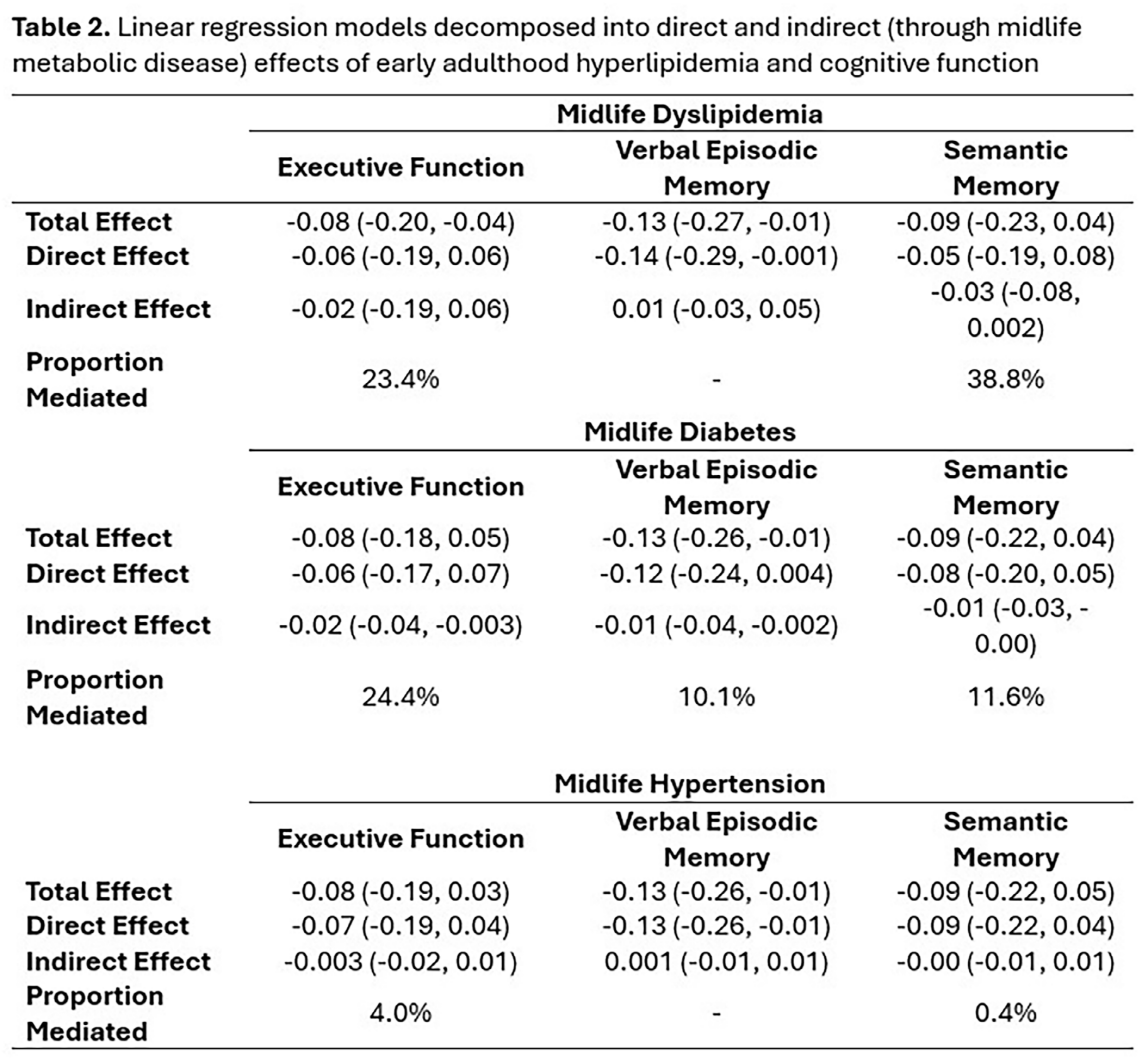# Mediating Role of Midlife Metabolic Disorders on the Association of Early Adulthood Hyperlipidemia and Late Life Cognition in Black Americans

**DOI:** 10.1002/alz70860_107208

**Published:** 2025-12-23

**Authors:** Kristen M. George, Paola Gilsanz, Rachel L. Peterson, Michelle M Mielke, Lisa L. Barnes, M. Maria Glymour, Charles DeCarli, Rachel A. Whitmer, Dan M. Mungas

**Affiliations:** ^1^ University of California, Davis, Davis, CA, USA; ^2^ Kaiser Permanente Northern California Division of Research, Pleasanton, CA, USA; ^3^ University of Montana, Missoula, MT, USA; ^4^ Division of Public Health Sciences, Wake Forest University, School of Medicine, Winston‐Salem, NC, USA; ^5^ Rush Alzheimer's Disease Center, Rush University Medical Center, Chicago, IL, USA; ^6^ Boston University School of Public Health, Boston, MA, USA; ^7^ University of California, Davis, Sacramento, CA, USA

## Abstract

**Background:**

Lipids play a vital role in brain function. Black Americans experience a disproportionate burden of midlife metabolic disorders and late‐life dementia despite, on average, favorable lipid profiles through midlife. In an all‐Black cohort, we aimed to assess the relationship between early adulthood hyperlipidemia and late‐life cognition, exploring midlife metabolic disorders as mediators.

**Method:**

This study included Black participants residing in northern California, USA, from the Study of Healthy Aging in African Americans and Kaiser Healthy Aging and Diverse Life Experiences Study. Early adulthood (mean age: 31.2±4.0) hyperlipidemia (total cholesterol ≥ 200 mg dL) was measured as part of routine care at Kaiser Permanente during Multiphasic Health Check‐ups (MHC) between ages 25‐40. Diagnoses of dyslipidemia, diabetes, and hypertension were ascertained from electronic health records in midlife (ages 40‐65 (mean diagnosis age range: 54‐57)). Cognition was assessed at baseline (ages 50+) using the Spanish and English Neuropsychological Assessment Scales measuring z‐standardized executive function (EF), verbal episodic memory (VEM), and semantic memory (SEM). Linear regression models associated early adulthood hyperlipidemia with late‐life cognition adjusting for gender, age at MHC, age at cognitive assessment, and education. Causal mediation analyses decomposed the total effect of early adulthood hyperlipidemia on cognition into natural direct effects and natural indirect effects via midlife metabolic disorders.

**Result:**

Participants (*n* = 773) had a mean total cholesterol of 198.6±39.4 mg dL and 44.5% prevalence of hyperlipidemia in early adulthood. Prevalence of midlife dyslipidemia was 46.2%, diabetes 23.9%, and hypertension 58.6%. Mean age at cognitive assessment was 74.3±6.6 years. Early adulthood hyperlipidemia was associated with significantly worse VEM (β[95%CI]:‐0.13[‐0.26,‐0.01]), and non‐significantly worse EF (β:‐0.07[‐0.19,0.04]) and SM (β:‐0.07[‐0.19, 0.04]). The association between early adulthood hyperlipidemia and EF was partially mediated by midlife dyslipidemia (23.4%), diabetes (24.4%), and hypertension (4.0%). The association with VEM was partially mediated by midlife diabetes (10.1%), but not dyslipidemia or hypertension. The association with SEM was mediated by dyslipidemia (38.8%) and diabetes (11.6%), but not hypertension.

**Conclusion:**

The associations of early adulthood hyperlipidemia on late‐life EF and SM may occur via vascular pathways partially mediated by midlife dyslipidemia and diabetes. Non‐vascular mechanisms should be examined between early adulthood hyperlipidemia and worse late‐life VEM.